# Impact of bronchoalveolar lavage lymphocytosis on the effects of anti-inflammatory therapy in idiopathic non-specific interstitial pneumonia, idiopathic pleuroparenchymal fibroelastosis, and unclassifiable idiopathic interstitial pneumonia

**DOI:** 10.1186/s12931-021-01726-8

**Published:** 2021-04-20

**Authors:** Akira Yamagata, Machiko Arita, Hiromasa Tachibana, Fumiaki Tokioka, Chikatoshi Sugimoto, Hiromitsu Sumikawa, Tomonori Tanaka, Hideki Yasui, Tomoyuki Fujisawa, Yutaro Nakamura, Takafumi Suda, Tadashi Ishida

**Affiliations:** 1 Department of Respiratory Medicine, Ohara Healthcare Foundation, Kurashiki Central Hospital, 1-1-1 Miwa, Kurashiki, Okayama 710-8602 Japan; 2 Department of Respiratory Medicine, Graduate School of Medicine, Kyoto University, 54 Kawahara-cho, Shogoin, Sakyo-ku, Kyoto, 606-8507 Japan; 3 Department of Respiratory Medicine, National Hospital Organization Minami Kyoto Hospital, 11 Nakaashihara, Joyo, Kyoto 610-0113 Japan; 4 Clinical Research Center, National Hospital Organization Kinki-Chuo Chest Medical Center, 1180 Nagasone-cho, Kita-ku, Sakai, Osaka 591-8555 Japan; 5 Department of Radiology, Sakai City Medical Center, 1-1-1 Ebaraji-cho, Nishi-ku, Sakai, Osaka 593-8304 Japan; 6 Department of Diagnostic Pathology, Kobe University Graduate School of Medicine, 7-5-2 Kusunoki-cho, Chuo-ku, Kobe, Hyogo 650-0017 Japan; 7 Second Division, Department of Internal Medicine, Hamamatsu University School of Medicine, 1-20-1 Handayama, Higashi-ku, Hamamatsu, Shizuoka 431-3192 Japan

**Keywords:** Bronchoalveolar lavage, Idiopathic interstitial pneumonia, Differential cell count

## Abstract

**Background:**

Idiopathic non-specific interstitial pneumonia (iNSIP), idiopathic pleuroparenchymal fibroelastosis (iPPFE), and unclassifiable idiopathic interstitial pneumonia (IIP) are IIPs with chronic fibrotic phenotypes, and unlike idiopathic pulmonary fibrosis, they have often been treated with anti-inflammatory drugs, including corticosteroids and immunosuppressants. However, the impact of bronchoalveolar lavage (BAL) lymphocytosis on the effects of anti-inflammatory therapy has never been evaluated. This study aimed to elucidate whether BAL lymphocytosis can be used to predict the efficacy of anti-inflammatory drugs for iNSIP, iPPFE, and unclassifiable IIP.

**Methods:**

Japanese patients diagnosed with iNSIP, iPPFE, and unclassifiable IIP by multidisciplinary discussion were identified using the nationwide registry. Eligible patients were stratified into four groups with and without BAL lymphocytosis and anti-inflammatory therapy to compare overall survival (OS) and changes in lung function. BAL lymphocytosis was defined as a lymphocyte differential count > 15%, and the cut-off was corroborated by survival classification and regression tree analysis.

**Results:**

Overall, 186 patients (37 iNSIP, 16 iPPFE, and 133 unclassifiable IIP) were analyzed. Limited to patients treated with anti-inflammatory drugs (n = 123), patients with BAL lymphocytosis had a better prognosis [hazard ratio (HR), 0.26; 95% confidence interval (CI), 0.11–0.63; *P* = 0.003], higher slope of forced vital capacity (FVC) % predicted for 2 years, and longer OS (log-rank test, *P* = 0.012) than those without BAL lymphocytosis. On multivariate analysis, BAL lymphocytosis (HR 0.31; 95% CI 0.13–0.75; *P* = 0.009) was a prognostic factor for OS, along with age and FVC % predicted. Conversely, for patients managed without anti-inflammatory therapy (n = 63), the presence or absence of BAL lymphocytosis had no prognostic value.

**Conclusions:**

BAL lymphocytosis is associated with good outcomes in patients treated with anti-inflammatory drugs, but has no prognostic value when anti-inflammatory drugs are not used. BAL lymphocytosis may provide a predictive biomarker for identifying patients with iNSIP, iPPFE and unclassifiable IIP who are likely to benefit from anti-inflammatory drugs.

**Supplementary Information:**

The online version contains supplementary material available at 10.1186/s12931-021-01726-8.

## Background

Idiopathic interstitial pneumonias (IIPs) are a group of interstitial lung diseases (ILDs) of unknown cause, and they are currently divided into nine types according to an international classification [1]. Of these, idiopathic pulmonary fibrosis (IPF) is the most common and shows a poor prognosis, with a median survival of about 3 years [2]. Corticosteroids and other immunosuppressants were widely used to treat IPF in the past, but these drugs have now been shown to offer no efficacy for IPF [3]. Anti-fibrotic drugs such as pirfenidone and nintedanib are now regarded as “conditionally” recommended drugs for IPF [4–6].

On the other hand, IIPs other than IPF are a heterogeneous group. Histological findings of cryptogenic organizing pneumonia (COP) and smoking-related idiopathic interstitial pneumonia, such as desquamative interstitial pneumonia (DIP) and respiratory bronchiolitis-associated ILD (RB-ILD), include cellular infiltration into the alveolar space or interstitium without overt evidence of fibrosis. The mainstay of pharmacotherapy for these diseases is corticosteroids [7, 8]. However, idiopathic non-specific interstitial pneumonia (iNSIP), idiopathic pleuroparenchymal fibroelastosis (iPPFE), and unclassifiable IIP are chronic fibrotic ILDs for which no definitive treatment regimens have yet been established. Although anti-inflammatory drugs such as corticosteroids and immunosuppressants are used depending on the case, responses to these therapies are highly variable. Nintedanib was recently shown to reduce decreases in forced vital capacity (FVC) in patients with progressive fibrosing ILD [9]. An ongoing phase II trial (RELIEF in LUNG FIBROSIS) is also investigating the efficacy and safety of pirfenidone for progressive, non-IPF lung fibrosis [10]. However, some cases remain refractory to anti-fibrotic therapies and are progressive. Which cases should be treated with anti-fibrotic drugs and which cases should be treated with anti-inflammatory drugs thus remain unclear.

According to the American Thoracic Society/European Respiratory Society (ATS/ERS) guidelines, bronchoalveolar lavage (BAL) lymphocytosis (lymphocytes > 15%) represents a lymphocytic cellular pattern such as COP or iNSIP [11], some cases of which have shown good recovery with anti-inflammatory drugs [1]. Similarly, a recent study demonstrated marginal FVC increases after initiation of corticosteroids in patients showing fibrotic HP with BAL lymphocytosis [12]. Patients with BAL lymphocytosis may thus be good candidates to receive anti-inflammatory drugs. However, the utility of cellular analysis of BAL in iNSIP, iPPFE, and unclassifiable IIP has not previously been evaluated in a multicenter clinical study. We hypothesized that evaluation of BAL lymphocytes would prove useful for predicting the response to anti-inflammatory drugs of patients with iNSIP, iPPFE, and unclassifiable IIP.

Testing this hypothesis, however, requires accurate diagnosis of iNSIP, iPPFE, and unclassifiable IIP. Since observers cannot discriminate specific ILD entities on the basis of clinical, radiological, or pathological data alone, accurate diagnosis of IIPs is often difficult [13]. Several studies have reported the importance of multidisciplinary discussion (MDD) to address such issues [14–16]. We recently developed a nationwide cloud-based integrated database to collect clinical, radiological, and pathological data from patients with biopsy-confirmed IIPs and a web-based MDD system to allow accurate establishment of IIP diagnoses [17].

The aim of this study was to clarify whether BAL lymphocytosis was associated with responsiveness to anti-inflammatory drugs in patients with iNSIP, iPPFE, and unclassifiable IIP through an analysis of a retrospective nationwide database by MDD.

## Methods

### Study design

A secondary analysis of a nationwide cloud-based integrated database along with a web-based MDD was performed retrospectively [17]. Complete details of the methods used to develop the database have been described previously [17]. The database was constructed from the records of Japanese patients diagnosed with IIPs in 39 institutions. Patients diagnosed at each institution with other ILDs such as hypersensitivity pneumonitis (HP) or connective tissue disease (CTD) ILD were excluded. All enrolled patients had undergone high-resolution computed tomography (HRCT) of the chest and surgical lung biopsy (SLB) between April 2009 and March 2014. Patients with acute exacerbations within 1 month after SLB were excluded. This study was approved by the institutional review boards of Kurashiki Central Hospital, Kurashiki, Japan (approval no. 3025) and Hamamatsu University School of Medicine, Hamamatsu, Japan (approval no. 19-042). The need for informed consent was waived due to the retrospective nature of the study.

### Inclusion and exclusion criteria

Cases of iNSIP, iPPFE, and unclassifiable IIP were identified from the online nationwide database, which was designed to include only patients with IIPs. Patients diagnosed with IPF, COP, DIP/RB-ILD, or ILD other than IIPs by MDD were excluded. Patients with insufficient BAL data were also excluded. Finally, 186 patients with iNSIP, iPPFE, and unclassifiable IIP having adequate BAL data were included in the analysis.

### Data collection and definitions

Baseline demographic, clinical, and HRCT data within 3 months before SLB were collected from the medical records. Results of blood tests, pulmonary function tests, and 6-min walking tests at 1 year (± 3 months) after SLB were obtained if available. The last pulmonary function tests during the observation period were also recorded. Following the ATS/ERS guideline [11], BAL was performed by wedging the tip of a fiberoptic bronchoscope into the selected bronchopulmonary segment, which was determined by respiratory physicians at each institution based on the extent of the interstitial shadows on HRCT and the expected recovery rate of the fluid. The total instilled volume of sterile saline (0.9% NaCl) was 100 or 150 ml. BAL lymphocytosis was defined as a lymphocyte differential count > 15%, in accordance with the guideline [11]. To corroborate that a cut-off of 15% for BAL lymphocytosis was appropriate for predicting prognosis, survival classification and regression tree (CART) analysis was used. The diagnosis at each institution was reported as the institutional diagnosis. The diagnosis made by MDD was categorized according to the ATS/ERS/JRS/Latin American Thoracic Association (ALAT) IPF statements [18] and the ATS/ERS IIPs classification [1]. HRCT and pathological features were also obtained from MDD. The imaging pattern was classified as a definite usual interstitial pneumonia (UIP) pattern, possible UIP pattern, or inconsistent with UIP pattern according to the ATS/ERS/JRS/ALAT IPF statements [18], because MDD was conducted before the 2018 IPF guideline was published [13]. Interstitial pneumonia with autoimmune features (IPAF) was defined according to the ERS/ATS research statement [19]. The treatment regimen given after SLB was determined by the respiratory physicians at each institution. Systemic corticosteroids or immunosuppressants were administered only after BAL and SLB were performed. As a 1-year outcome, improvement was defined as a ≥ 10% increase in FVC % predicted or a ≥ 15% increase in diffusion capacity for carbon monoxide (DL_CO_) % predicted, and worsening was defined as a > 10% decrease in FVC % predicted or a > 15% decrease in DL_CO_ % predicted. Overall survival (OS) was estimated from the date of SLB to death or last follow-up. The vital status of each patient was ascertained as of October 2017 for survival analysis.

### Statistical analysis

Continuous variables are expressed as means and standard deviations, and categorical variables are expressed as frequencies and percentages, as appropriate. Continuous variables were analyzed using Student’s *t*-test, and categorical variables were analyzed using the chi-squared test or Fisher’s exact test. Correlations between BAL lymphocytes and interstitial cell infiltration on pathological examination were assessed using Spearman’s test and the Jonckheere-Terpstra trend test. Survival probability was assessed using Kaplan–Meier methods and compared by log-rank testing. Multivariate analyses using Cox regression were conducted to identify variables associated with mortality. Results of Cox proportional hazards analyses are reported as hazard ratios (HRs) with 95% confidence intervals (CIs). Slopes of FVC % predicted were compared between patients with or without BAL lymphocytosis using linear mixed-effects models. Significance was defined at the level of *P* < 0.05. All analyses were performed using R version 3.6.1 software (The R Foundation for Statistical Computing, Vienna, Austria).

## Results

### Study population and comparisons between groups

**Fig. 1:**
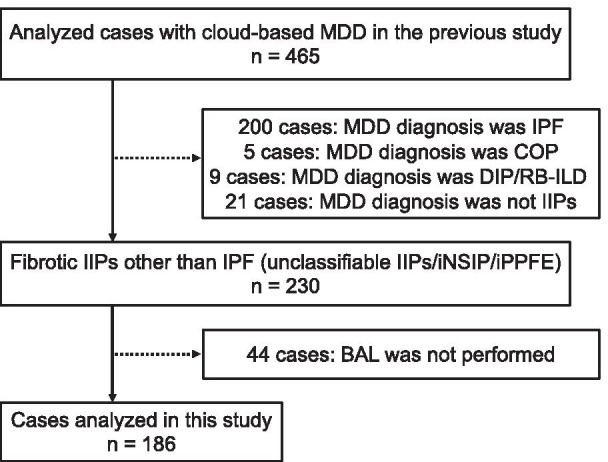
Study flowchart. *BAL* bronchoalveolar lavage, *COP* cryptogenic organizing pneumonia, *CTD-ILD* connective tissue disease-associated interstitial lung disease, *DIP* desquamative interstitial pneumonia, *HP* hypersensitivity pneumonia, *IIP* idiopathic interstitial pneumonia, *iNSIP* idiopathic non-specific interstitial pneumonia, *IPF* idiopathic pulmonary fibrosis, *iPPFE* idiopathic pleuroparenchymal fibroelastosis, *MDD* multidisciplinary discussion, *RB-ILD* respiratory bronchiolitis-associated interstitial lung disease. Of the 465 cases analyzed in the previous study [17], 200 cases diagnosed with IPF, 5 cases diagnosed with COP, and 9 cases diagnosed with DIP/RB-ILD by MDD were excluded. Twenty-one cases diagnosed with ILD other than IIPs [HP (n = 12), CTD-ILD (n = 5), lymphoproliferative disease (n = 3), and invasive mucinous adenocarcinoma (n = 1)] were also excluded. Of the 230 patients with fibrotic IIPs other than IPF, 44 cases for whom BAL was not performed were excluded. Finally, 186 patients were included for analysis

Figure 1 shows the flow diagram for the process of subject selection in this study. Of the 465 patients analyzed in the previous study, patients diagnosed with IPF (n = 200), COP (n = 5), DIP/RB-ILD (n = 9), or ILD other than IIPs (n = 21) were excluded. Of the 230 patients with iNSIP, iPPFE, and unclassifiable IIP, 44 cases for whom BAL was not performed were excluded. Finally, 186 patients (37 iNSIP, 16 iPPFE, and 133 unclassifiable IIP) were enrolled in the present analysis. The reasons for patients being unclassified are shown in Additional file 1: Table S1. Ninety-three patients (69.9%) had overlapping histological features, 20 patients (15.0%) had major discrepancies among clinical, radiological, and histological features, 11 patients (8.3%) had uncertain etiology, and 9 patients (6.8%) had inadequate clinical, radiological, or pathological data. The study population comprised 116 men (62.4%) and 70 women (37.6%), with a mean age of 62.5 ± 10.5 years. Of these, 123 (66.1%) were treated with anti-inflammatory drugs. The baseline characteristics of the enrolled population and comparisons between patients with and without anti-inflammatory therapy are shown in Table 1. Fifty-one patients (27.4%) were diagnosed with IPF as the institutional diagnosis. Fifty-five patients (29.6%) fit the definition for a definite or possible UIP pattern on HRCT, and 105 patients (51.3%) fit a broader definition of pathological UIP pattern. Female sex (*P* = 0.047), iNSIP (*P* = 0.006), and IPAF (*P* < 0.001) were more common among patients treated with anti-inflammatory drugs than among those managed without anti-inflammatory drug therapy. In addition, compared with patients managed without anti-inflammatory drug therapy, those managed with anti-inflammatory drug therapy showed more severe impairment of FVC % predicted (*P* = 0.025), DL_CO_ % predicted (*P* = 0.006), and PaO_2_/FiO_2_ ratio (*P* = 0.014), and increased serum levels of Krebs von den Lungen-6 (KL-6) (*P* = 0.020) and surfactant protein-D (SP-D) (*P* = 0.011). No significant differences in respiratory symptoms or the 6-min walking test were apparent between the groups.

**Table 1:** Baseline characteristics

	Anti-inflammatory drugs		*P* value	*n*
	No (n = 63)	Yes (n = 123)		
Male sex	46 (73.0)	70 (56.9)	0.047	186
Age (year)	64.0 ± 9.3	61.7 ± 11.1	0.135	186
Never-smoker	27 (43.6)	59 (48.8)	0.609	183
Modified MRC				
0	25 (47.2)	32 (28.3)	0.117	166
1	21 (39.6)	64 (56.6)		
2	6 (11.3)	14 (12.4)		
3	1 (1.9)	3 (2.7)		
4	0 (0.0)	0 (0.0)		
6-min walk test				
Distance (m)	514.6 ± 152.9	476.1 ± 137.3	0.148	137
Minimum SpO_2_ (%)	90.0 ± 5.5	89.2 ± 6.1	0.443	135
Respiratory function				
FVC % predicted (%)	83.9 ± 21.9	76.7 ± 17.5	0.025	185
DL_CO_ % predicted (%)	78.9 ± 24.5	64.3 ± 22.8	0.006	162
Laboratory examinations				
LDH (IU/L)	225.4 ± 81.8	242.2 ± 67.7	0.162	186
KL-6 (U/mL)	1331 ± 1255	1874 ± 1873	0.020	186
SP-D (ng/mL)	219.5 ± 176.3	316.0 ± 296.6	0.011	162
PaO_2_/FiO_2_ ratio (mmHg)	410.8 ± 67.5	385.7 ± 50.1	0.014	174
BAL				
Total volume of retrieved fluid (%)	50.3 ± 12.2	48.7 ± 15.4	0.470	177
Total cell count (× 10^5^/mL)	2.0 ± 1.6	2.7 ± 2.0	0.012	178
Neutrophils (%)	5.0 ± 11.9	6.2 ± 10.7	0.510	186
Lymphocytes (%)	18.0 ± 19.4	20.9 ± 19.7	0.340	186
Eosinophils (%)	3.1 ± 4.5	3.8 ± 5.5	0.365	186
Macrophages (%)	73.6 ± 24.1	69.4 ± 24.5	0.266	186
CD4^+^/CD8^+^ ratio	2.0 ± 1.9	1.7 ± 1.9	0.388	180
HRCT pattern in MDD				
Definite UIP	0 (0.0)	4 (3.3)	0.004	186
Possible UIP	26 (41.3)	25 (20.3)		
Inconsistent with UIP	37 (58.7)	91 (76.4)		
Pathological pattern				
Definite UIP	1 (1.7)	6 (4.0)	0.298	181
Probable UIP	14 (23.3)	30 (24.8)		
Possible UIP	23 (38.3)	31 (25.6)		
Not UIP	22 (36.7)	54 (44.6)		
Pathological features (none/mild/moderate/prominent)				
Fibroblastic foci	20/27/13/0	46/41/31/2	0.424	180
Honeycombing	44/11/5/0	86/22/9/1	0.911	178
Organizing pneumonia	45/10/4/1	65/18/24/11	0.013	178
Bronchiolocentric fibrosis	27/26/6/0	76/35/4/0	0.020	174
Lymphoid follicle	20/31/7/1	24/67/25/3	0.167	178
Alveolar macrophages	11/33/15/0	15/80/25/0	0.346	179
Interstitial cell infiltration	4/44/8/2	9/62/43/6	0.013	178
Bronchiolitis	30/24/4/0	65/43/9/0	0.837	175
Fibroelastosis	45/7/4/3	95/16/4/6	0.762	180
MDD diagnosis				
iNSIP	5 (7.9)	32 (26.0)	0.006	186
iPPFE	8 (12.7)	8 (6.5)		
Unclassifiable IIP	50 (79.4)	83 (67.5)		
IPAF	8 (12.7)	51 (41.5)	< 0.001	186

*BAL* bronchoalveolar lavage, *COP* cryptogenic organizing pneumonia, *DL*_*CO*_ diffusion capacity for carbon monoxide, *FEV*_*1*_ forced expiratory volume in 1 s, *FiO*_*2*_ fractional concentration of oxygen in inspired gas, *FVC* forced vital capacity, *HRCT* high-resolution computed tomography, *IIP* idiopathic interstitial pneumonia, *IPAF* interstitial pneumonia with autoimmune features, *IPF* idiopathic pulmonary fibrosis, *KL-6* Krebs von den Lungen-6, *LDH* lactate dehydrogenase, *MDD* multidisciplinary discussion, *MRC* Medical Research Council, *iNSIP* idiopathic non-specific interstitial pneumonia, *PaO*_*2*_ partial pressure of oxygen in arterial blood, *iPPFE* idiopathic pleuroparenchymal fibroelastosis, *SLB* surgical lung biopsy, *SP-D* surfactant protein D, *SpO*_*2*_ percutaneous oxyhemoglobin saturation, *TBE* traction bronchiectasis, *UIP* usual interstitial pneumonia

### Correlation between BAL lymphocytes and interstitial cell infiltration on histopathological examination

**Fig. 2:**
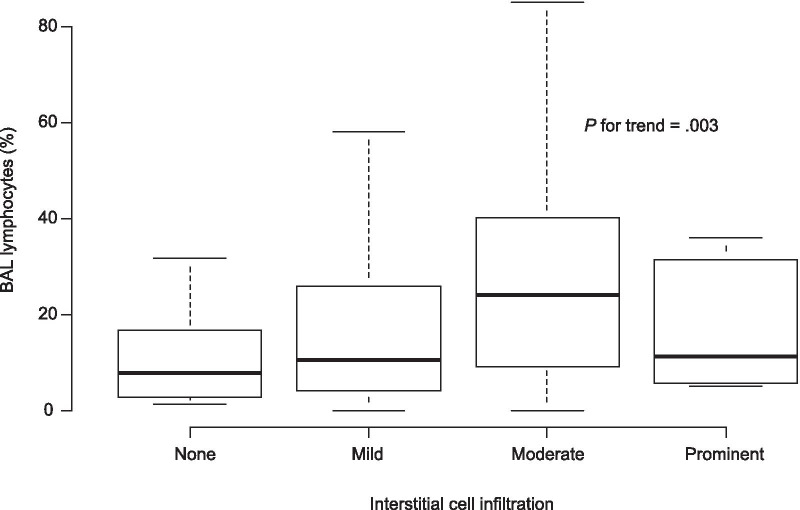
Correlation between BAL lymphocytes and interstitial cell infiltration on histopathological examination. *BAL* bronchoalveolar lavage. In the whole population, a significant positive correlation is apparent between BAL lymphocytes and interstitial cell infiltration (r = 0.231, P for trend = 0.003)

Correlations between BAL lymphocytes and interstitial cell infiltration on histopathological examination are shown in Fig. 2. A significant positive correlation was observed between BAL lymphocytes and interstitial cell infiltration in the whole population (r = 0.231, P for trend = 0.003). In a subgroup of patients who did not receive anti-inflammatory drugs, there was no significant correlation between BAL lymphocytes and interstitial cell infiltration (r = 0.156, P for trend = 0.250) (Additional file 3: Figure S1).

### Treatments and outcomes

Treatments and outcomes are shown in Table 2. In patients treated with anti-inflammatory drugs, 39 (31.7%) received corticosteroid monotherapy, and 84 (68.3%) received combination therapy comprising corticosteroids and other immunosuppressants. No significant differences in the use of anti-fibrotic drugs were seen between the groups. Patients treated with anti-inflammatory drugs showed a greater decrease in SP-D (*P* = 0.004), a greater increase in FVC % predicted (*P* = 0.016), and better outcomes (*P* = 0.015) after 1 year than patients managed without anti-inflammatory therapy. Forty-two patients (22.6%) died during the observation period. Of the deceased patients, 31 (73.8%) had received anti-inflammatory drugs. The most common cause of death was chronic respiratory failure (40.5%), followed by acute exacerbation (21.4%) and respiratory infection (16.7%). No significant differences in causes of death were seen between the groups. The mean follow-up period was 4.4 ± 2.3 years.

**Table 2:** Treatments and outcomes of patients

	Anti-inflammatory drugs		*P* value	*n*
	No (n = 63)	Yes (n = 123)		
Corticosteroid monotherapy	0 (0.0)	39 (31.7)	< 0.001	186
Combined with immunosuppressants	0 (0.0)	84 (68.3)	< 0.001	186
Cyclosporine A	0 (0.0)	56 (45.5)		
Tacrolimus	0 (0.0)	5 (4.1)		
Cyclophosphamide	0 (0.0)	13 (10.6)		
Azathioprine	0 (0.0)	10 (8.1)		
Anti-fibrotic drugs*	20 (31.7)	30 (24.4)	0.370	186
Pirfenidone	14 (22.2)	23 (18.7)		
Nintedanib	5 (7.9)	7 (5.7)		
One-year changes				
ΔLDH (IU/L)	− 15.1 ± 84.7	− 16.8 ± 192.3	0.934	168
ΔKL-6 (U/mL)	− 347 ± 1234	− 602 ± 1691	0.270	169
ΔSP-D (ng/mL)	16.4 ± 121.1	− 168.5 ± 558.1	0.004	131
ΔFVC % predicted (%)	− 2.9 ± 10.9	3.1 ± 19.7	0.016	156
ΔDL_CO_ % predicted (%)	− 1.9 ± 25.6	5.5 ± 20.8	0.113	132
One-year outcomes				
Improvement**	7 (14.9)	41 (37.6)	0.015	156
Stable	26 (55.3)	44 (40.4)		
Worsening***	14 (29.8)	24 (22.0)		
Death during observation period	11 (17.5)	31 (25.2)	0.270	186

*One patient with missing data on which of the anti-fibrotic drugs was used

**Improvement was defined as a ≥ 10% increase in %FVC or a ≥ 15% increase in %DL_CO_

***Worsening was defined as a > 10% decrease in %FVC or a > 15% decrease in %DL_CO_

*DL*_*CO*_ diffusion capacity for carbon monoxide, *FVC* forced vital capacity, *KL-6* Krebs von den Lungen-6, *LDH* lactate dehydrogenase, *SP-D* surfactant protein D

### Corroboration of the validity of the 15% cut-off value for BAL lymphocytosis

Whether BAL lymphocytosis was a prognostic factor in patients treated with anti-inflammatory drugs was investigated by varying the cut-off value. Patients with BAL lymphocytosis tended to consistently have a better prognosis at almost any cut-off value, and BAL lymphocytosis was found to be a significant factor related to a good prognosis with a cut-off value of 9–19% (Additional file 4: Figure S2). Based on CART analysis for prediction, the optimal cut-off value of BAL lymphocytosis was 16.6% in this population (Additional file 5: Figure S3). Only six patients showed BAL lymphocytes between 15% and 16.6% in the present study. These results support the previous guideline that BAL lymphocytosis (lymphocytes > 15%) represents a lymphocytic cellular pattern [11]. Thus, BAL lymphocytosis was defined as a lymphocyte differential count > 15%. In the present study, 79 patients (42.5%) showed BAL lymphocytosis. There were no significant differences in environmental exposures, symptoms suggestive of CTD, or serum autoantibodies associated with CTD between the two groups, which were divided by the presence or absence of BAL lymphocytosis (Additional file 2: Table S2).

### Impact of BAL lymphocytosis on response to anti-inflammatory drugs

**Fig. 3:**
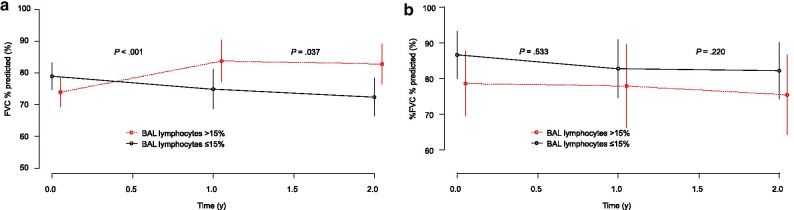
Slope of FVC % predicted for 2 years after SLB. *BAL* bronchoalveolar lavage, *FVC* forced vital capacity, *SLB* surgical lung biopsy. For patients treated with anti-inflammatory drugs, patients with BAL lymphocytosis show a higher slope of FVC % predicted than patients without BAL lymphocytosis (**a**). On the other hand, limited to patients without anti-inflammatory drugs, no difference is evident between the groups (**b**)

**Fig. 4:**
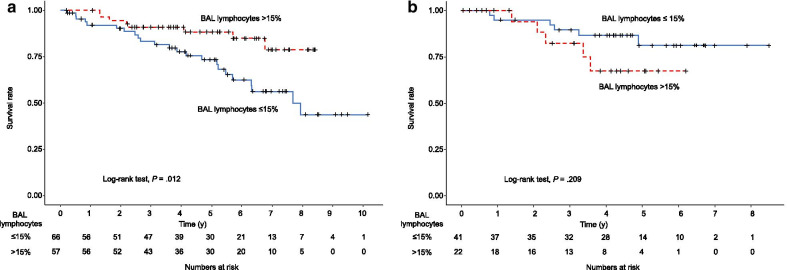
Kaplan–Meier plot of survival probability. *BAL* bronchoalveolar lavage, *IPF* idiopathic pulmonary fibrosis, *OS* overall survival. For patients treated with anti-inflammatory drugs, patients with BAL lymphocytosis have longer OS than those without BAL lymphocytosis (log-rank test, *P* = .012) (**a**). On the other hand, limited to patients without anti-inflammatory drugs, no significant difference in OS is seen between patients with and without BAL lymphocytosis (log-rank test, *P* = 0.209) (**b**)

Table 3 shows HRs for OS in each group, divided by the presence or absence of anti-inflammatory drugs and BAL lymphocytosis. Limited to patients treated with anti-inflammatory drugs, patients with BAL lymphocytosis had a better prognosis than patients without BAL lymphocytosis (HR 0.26; 95% CI 0.11–0.63; *P* = 0.003). On the other hand, in patients managed without anti-inflammatory therapy, those with BAL lymphocytosis tended to have a worse prognosis than those without BAL lymphocytosis, though the difference was not significant (HR 1.88; 95% CI 0.54–6.56; *P* = 0.322). Figure 3 shows the slope of FVC % predicted for 2 years after SLB. For patients treated with anti-inflammatory drugs, patients with BAL lymphocytosis had a higher slope of FVC % predicted than patients without BAL lymphocytosis (Fig. 3a). On the other hand, when limited to patients managed without anti-inflammatory drugs, no difference between groups was evident (Fig. 3b). On Kaplan–Meier analysis of patients managed with anti-inflammatory drugs, patients with BAL lymphocytosis had longer OS than those without BAL lymphocytosis (log-rank test, *P* = 0.012) (Fig. 4a). Conversely, limited to patients managed without anti-inflammatory therapy, no significant difference in OS was noted between patients with and without BAL lymphocytosis (log-rank test, *P* = 0.209) (Fig. 4b). Multivariate analysis of patients managed with anti-inflammatory drugs showed that BAL lymphocytosis (HR 0.31; 95% CI 0.13–0.75; *P* = 0.009), age (HR 1.04; 95% CI 1.01–1.08; *P* = 0.022), and FVC % predicted (HR, 0.95; 95% CI 0.93–0.98; *P* < 0.001) were significant prognostic factors for OS (Table 4).

**Table 3:** Hazard ratios for overall survival

	BAL lymphocytes	
	≤ 15% (n = 107)	> 15% (n = 79)
Anti-inflammatory drugs		
Yes (n = 123)	1.00	0.26 (0.11–0.63)
No (n = 63)	0.43 (0.17–1.08)	0.80 (0.29–2.18)

*BAL* bronchoalveolar lavage

**Table 4:** Multivariate Cox regression analysis of overall survival in patients treated with anti-inflammatory drugs

	Multivariate analysis	
	HR (95% CI)	*P* value
Age (year)	1.04 (1.01–1.08)	0.022
Male	2.13 (0.94–4.79)	0.068
FVC % predicted (%)	0.95 (0.93–0.98)	< 0.001
BAL lymphocytosis	0.31 (0.13–0.75)	0.009
Anti-fibrotic drugs	1.94 (0.90–4.19)	0.090

*BAL* bronchoalveolar lavage, *CI* confidence interval, *FVC* forced vital capacity, *HR* hazard ratio

## Discussion

This is the first multicenter, clinical study involving a cellular analysis of BAL in patients with iNSIP, iPPFE, and unclassifiable IIP. The results showed that BAL lymphocytosis was associated with improvement of FVC % predicted and was a prognostic factor for good OS in patients with anti-inflammatory drugs, whereas BAL lymphocytosis proved to be unrelated to prognosis when anti-inflammatory drugs were not used. These results suggest that BAL lymphocytosis is not a prognostic factor in itself, but instead represents a predictive biomarker for response to anti-inflammatory drugs. BAL lymphocytosis may thus help identify patients for whom anti-inflammatory therapy is likely to prove beneficial. On the assumption that inflammation causes progressive fibrosis and that anti-inflammatory drugs ameliorate these effects by reducing the numbers of inflammatory cells in the lungs, measurement of inflammatory cells in BAL has become common practice for identifying patients with IIPs who warrant anti-inflammatory drugs. Indeed, BAL lymphocytosis is reportedly associated with a better prognosis in fibrotic IIPs and iNSIP [20, 21]. In the present study, BAL lymphocytes were positively correlated with interstitial cell infiltration on histopathological examination in the whole population, indicating that BAL lymphocytosis may reflect increased numbers of inflammatory cells in alveolar tissue. In many cases, SLB cannot be performed due to its high invasiveness. BAL is a minimally invasive procedure and represents a potential alternative for predicting response to anti-inflammatory drugs in patients who cannot undergo SLB.

There are conditional recommendations for the use of anti-fibrotic drugs in IPF [4–6]. The main benefit of these drugs is that they slow the rate of decline in pulmonary function. The INBUILD trial recently showed that nintedanib reduced FVC decreases in patients with progressive fibrosing ILD [9]. Subgroup analyses in the INBUILD trial also demonstrated the efficacy of nintedanib for iNSIP and unclassifiable IIP [22]. Furthermore, a recent retrospective study showed the efficacy and safety of anti-fibrotic drugs for progressive fibrosing non-IPF ILDs [23]. The proven effects of an anti-fibrotic drug for reducing FVC decreases in progressive fibrosing ILD in the INBUILD trial are important, showing another treatment option. However, the protocol in that trial did not allow for use of anti-inflammatory drugs for 6 months after randomization. A subgroup of patients may exist in whom efficacy of anti-inflammatory drugs can also be expected. Patients with BAL lymphocytosis had good outcomes for iNSIP, iPPFE, and unclassifiable IIP treated with anti-inflammatory drugs in the present study, suggesting that some patients with progressive fibrosing ILD respond to anti-inflammatory drugs. As shown in a clinical trial for systemic sclerosis-associated ILD [24], appropriate use of anti-fibrotic and anti-inflammatory drugs in selected patients with iNSIP, iPPFE, and unclassifiable IIP is expected to improve prognosis.

In the present study, the most prevalent MDD diagnosis was unclassifiable IIP (71.5%). The main reasons for IIPs being unclassifiable after SLB are considered to be as follows: (1) overlapping histological features; (2) major discrepancies among clinical, radiological, and histological features; and (3) uncertain etiology. In the present study population, the majority of patients with unclassifiable IIP had overlapping histological features, most of which represented UIP and NSIP. Although a recent phase II trial suggested the efficacy of pirfenidone for progressive fibrosing unclassifiable IIP [25], identifying the most appropriate therapy is particularly challenging given the diverging treatment approaches for IIPs. In the present study, BAL lymphocytosis appeared to be a useful biomarker for predicting response to anti-inflammatory therapy, instead of radiological or histological features. Benefits of anti-inflammatory approaches may thus be expected in some patients with unclassifiable IIP, especially with BAL lymphocytosis.

Most of the patients who participated in the present study were screened for a history of environmental exposures, symptoms suggestive of CTD, and serum autoantibodies associated with CTD, although data on precipitating antibodies for HP were not collected. Moreover, all patients had undergone SLB and BAL and were diagnosed through MDD, contributing to the reliability of the diagnosis. However, low levels of inter-MDD agreement for ILDs were observed even among expert centers due to diagnostic heterogeneity, and, in particular, the absence of consensus guidelines for the diagnosis of fibrotic HP has resulted in substantial variability and lack of reproducibility in the assignment of an HP diagnosis [26, 27]. To overcome the lack of consistency in diagnostic approach, an international working group proposed an ontological framework, in which patients with fibrotic ILD were categorized as confident diagnosis, “provisional” diagnosis, or “unclassifiable ILD” according to their diagnostic likelihood [28]. Since strict adherence to diagnostic criteria may have resulted in a high proportion of unclassifiable IIP in our original database, this standardized diagnostic ontology could have made our study more generalizable. However, this document was published after our database was constructed and could not be applied to our MDD, which is a major limitation of the present study.

There are other limitations in this study that should be noted. First, the study was conducted retrospectively, and some data, especially in terms of disease behavior until SLB, were lacking. Thus, pulmonary function trends before and after anti-inflammatory therapy could not be assessed in the same population. Second, given that the enrolled population included multiple disease entities, the present study might have included some degree of selection bias for anti-inflammatory therapy. A patient population that could be more likely to benefit from anti-inflammatory drugs might have been selected for the treatment group. Indeed, patients treated with anti-inflammatory drugs showed a higher proportion of female patients, and higher rates of iNSIP and IPAF, which are often treated with anti-inflammatory drugs [29]. Third, this study was conducted only with patients who had undergone SLB, so the present results are not necessarily applicable to patients who have not undergone SLB. However, as mentioned above, a significant positive correlation was observed between BAL lymphocytes and interstitial cell infiltration in the present study. BAL lymphocytes, which could reflect pathological findings, might offer a useful biomarker even for patients who have not undergone SLB. Finally, treatment regimens such as the dose and period of corticosteroid administration and types of immunosuppressants varied from case to case. Further prospective multicenter examinations should be conducted to evaluate the impact of BAL lymphocytosis on response to anti-inflammatory drugs in patients with iNSIP, iPPFE, and unclassifiable IIP.

## Conclusions

The present data suggest that BAL lymphocytosis is associated with good outcomes in patients treated with anti-inflammatory drugs, whereas BAL lymphocytosis showed no association with prognosis in patients managed without anti-inflammatory therapy. BAL lymphocytosis appears to be a predictive biomarker identifying patients with iNSIP, iPPFE, and unclassifiable IIP who are likely to benefit from anti-inflammatory drugs.

## Supplementary Information

**Additional file 1:** **Table S1.** Reasons for IIPs being unclassified in MDD.

**Additional file 2: Table S2.** Environmental exposures, symptoms suggestive of CTD, and serologic markers associated with CTD

**Additional file 3: Fig. S1.** Correlation between BAL lymphocytes and interstitial cell infiltration on histopathological examination in a subgroup of patients without anti-inflammatory drugs.

**Additional file 4: Fig. S2.** Prognostic impact of BAL lymphocytosis on patients treated with anti-inflammatory drugs at each cut-off value

**Additional file 5: Fig. S3.** CART analysis for predicting prognosis.

## Data Availability

The datasets used and/or analyzed during the current study are available from the corresponding author on reasonable request.

## Abbreviations

ATS: American Thoracic Society
BAL: Bronchoalveolar lavage
CART: Classification and regression tree
CI: Confidence interval
COP: Cryptogenic organizing pneumonia
CTD: Connective tissue disease
DIP: Desquamative interstitial pneumonia
DL_CO_: Diffusion capacity for carbon monoxide
ERS: European Respiratory Society
FEV_1_: Forced expiratory volume in 1 s
FVC: Forced vital capacity
HP: Hypersensitivity pneumonitis
HR: Hazard ratio
HRCT: High-resolution computed tomography
IIPs: Idiopathic interstitial pneumonias
iNSIP: Idiopathic non-specific interstitial pneumonia
IPF: Idiopathic pulmonary fibrosis
iPPFE: Idiopathic pleuroparenchymal fibroelastosis
KL-6: Krebs von den Lungen-6
LDH: Lactate dehydrogenase
MDD: Multidisciplinary discussion
mMRC: Modified Medical Research Council
OS: Overall survival
PaO_2_: Partial pressure of oxygen in arterial blood
RB-ILD: Respiratory bronchiolitis-interstitial lung disease
SLB: Surgical lung biopsy
SP-D: Surfactant protein-D
SpO_2_: Percutaneous oxyhemoglobin saturation
TBE: Traction bronchiectasis
UIP: Usual interstitial pneumonia
