# Supplementary material for: Impact of bronchoalveolar lavage lymphocytosis on the effects of anti-inflammatory therapy in idiopathic non-specific interstitial pneumonia, idiopathic pleuroparenchymal fibroelastosis, and unclassifiable idiopathic interstitial pneumonia
Source: Respir Res. 2021 Apr 20;22:115. doi: 10.1186/s12931-021-01726-8 (PMC8059166; doi:10.1186/s12931-021-01726-8)
Supplement: Supplementary file 1 — Additional file 1: Table S1. Reasons for IIPs being unclassified in MDD. [file 12931_2021_1726_MOESM1_ESM.docx]

**Table S1.** Reasons for IIPs being unclassified in MDD

|  | Anti-inflammatory drugs | |
| --- | --- | --- |
|  | No (n = 50) | Yes (n = 83) |
| Overlapping histological features |  |  |
| UIP+PPFE | 0 | 8 |
| UIP+PPFE+NSIP | 0 | 1 |
| UIP+NSIP | 20 | 25 |
| UIP+NSIP+OP | 0 | 2 |
| UIP+NSIP+DIP | 0 | 3 |
| UIP+OP | 2 | 2 |
| UIP+DIP | 1 | 0 |
| NSIP+OP | 7 | 19 |
| NSIP+DIP | 2 | 1 |
| Major discrepancies among clinical, radiological, and histological features | 9 | 11 |
| Uncertain etiology | 5 | 6 |
| Inadequate clinical, radiological, or pathological data | 4 | 5 |

*Abbreviations*: DIP, desquamative interstitial pneumonia; OP, organizing pneumonia; IIP, idiopathic interstitial pneumonia; MDD, multidisciplinary discussion; NSIP, non-specific interstitial pneumonia; PPFE, pleuroparenchymal fibroelastosis; UIP, usual interstitial pneumonia.
