# Supplementary material for: Impact of bronchoalveolar lavage lymphocytosis on the effects of anti-inflammatory therapy in idiopathic non-specific interstitial pneumonia, idiopathic pleuroparenchymal fibroelastosis, and unclassifiable idiopathic interstitial pneumonia
Source: Respir Res. 2021 Apr 20;22:115. doi: 10.1186/s12931-021-01726-8 (PMC8059166; doi:10.1186/s12931-021-01726-8)
Supplement: Supplementary file 2 — Additional file 2: Table S2. Environmental exposures, symptoms suggestive of CTD, and serologic markers associated with CTD [file 12931_2021_1726_MOESM2_ESM.docx]

**Table S2.** Environmental exposures, symptoms suggestive of CTD, and serologic markers associated with CTD

|  | BAL lymphocytes | | *P* value | *n* |
| --- | --- | --- | --- | --- |
|  | ≤15% (n = 107) | >15% (n = 79) |  |  |
| Environmental exposure | 23 (22.1) | 13 (18.3) | .574 | 175 |
| Asbestos | 7 (6.7) | 3 (4.2) |  |  |
| Organic matter | 7 (6.7) | 5 (7.0) |  |  |
| Inorganic matter | 9 (8.7) | 5 (7.0) |  |  |
| Symptoms suggestive of CTD | 16 (15.2) | 14 (19.4) | .542 | 177 |
| Distal digital fissuring | 1 (1.0) | 3 (4.4) |  |  |
| Distal digital tip ulceration | 1 (1.0) | 0 (0.0) |  |  |
| Inflammatory arthritis or polyarticular morning joint stiffness ≥60 min | 4 (3.9) | 6 (8.5) |  |  |
| Palmar telangiectasia | 3 (3.2) | 1 (1.5) |  |  |
| Raynaud’s phenomenon | 7 (6.8) | 2 (2.9) |  |  |
| Unexplained digital edema | 3 (3.1) | 3 (4.2) |  |  |
| Gottron’s sign | 2 (2.0) | 1 (1.5) |  |  |
| Serologic markers associated with CTD | 27 (25.2) | 27 (34.2) | .195 | 186 |
| Antinuclear antibody titer ≥320, nucleolar pattern or centromere pattern | 12 (12.2) | 10 (15.2) |  | 164 |
| Rheumatoid factor ≥2× upper limit of normal | 12 (12.2) | 10 (15.2) |  | 164 |
| Anti-CCP | 2 (2.8) | 2 (4.0) |  | 122 |
| Anti-dsDNA | 3 (4.4) | 0 (0.0) |  | 102 |
| Anti-SS-A | 4 (4.2) | 5 (7.4) |  | 164 |
| Anti-SS-B | 0 (0.0) | 0 (0.0) |  | 160 |
| Anti-ribonucleoprotein | 0 (0.0) | 0 (0.0) |  | 143 |
| Anti-Smith | 2 (2.9) | 1 (2.9) |  | 104 |
| Anti-topoisomerase | 3 (3.1) | 1 (1.6) |  | 160 |
| Anti-tRNA synthetase | 2 (1.9) | 4 (5.8) |  | 172 |
| Anti-PM-Scl | 0 (0.0) | 0 (0.0) |  | 13 |
| Anti-MDA-5 | 0 (0.0) | 0 (0.0) |  | 5 |
| IPAF | 29 (27.1) | 30 (38.0) | .151 | 186 |

*Abbreviations*: BAL, bronchoalveolar lavage; CTD, connective tissue disease; IPAF, interstitial pneumonia with autoimmune features.
