# Supplementary figures and images for: Impact of bronchoalveolar lavage lymphocytosis on the effects of anti-inflammatory therapy in idiopathic non-specific interstitial pneumonia, idiopathic pleuroparenchymal fibroelastosis, and unclassifiable idiopathic interstitial pneumonia
Source: Respir Res. 2021 Apr 20;22:115. doi: 10.1186/s12931-021-01726-8 (PMC8059166; doi:10.1186/s12931-021-01726-8)

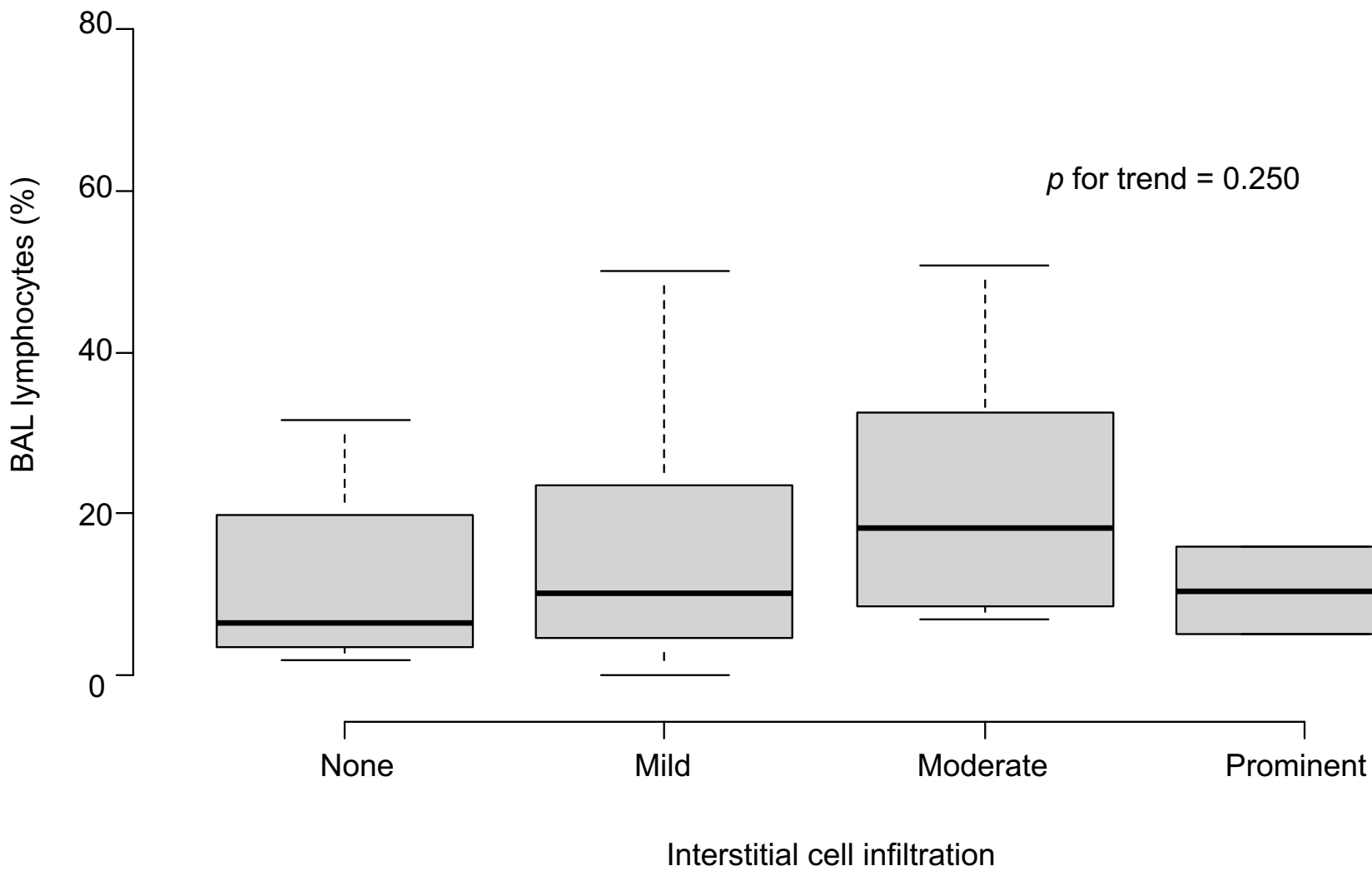

Supplement: Supplementary file 3 — Additional file 3: Fig. S1. Correlation between BAL lymphocytes and interstitial cell infiltration on histopathological examination in a subgroup of patients without anti-inflammatory drugs. [file 12931_2021_1726_MOESM3_ESM.pdf]

A)

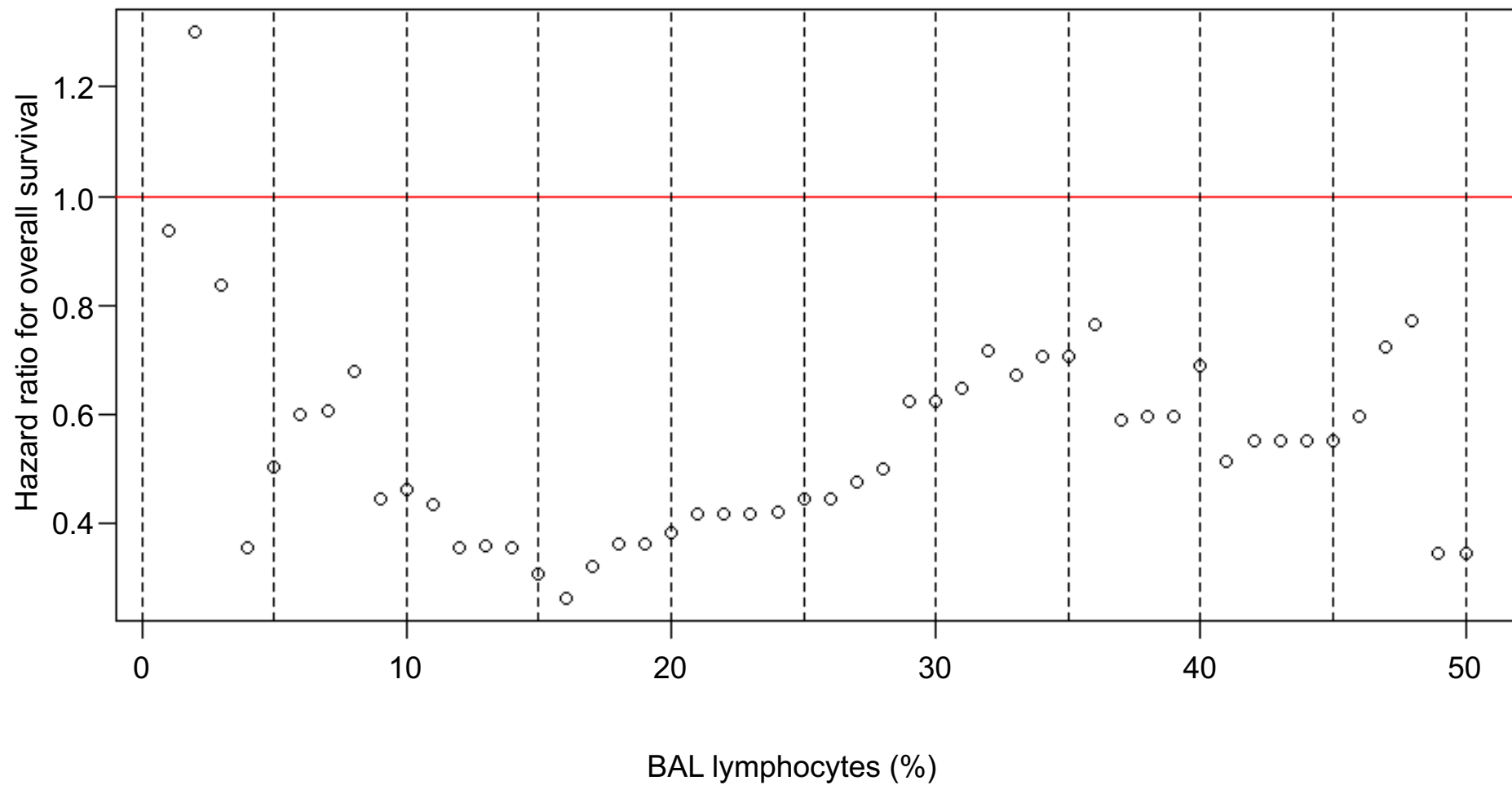

B)

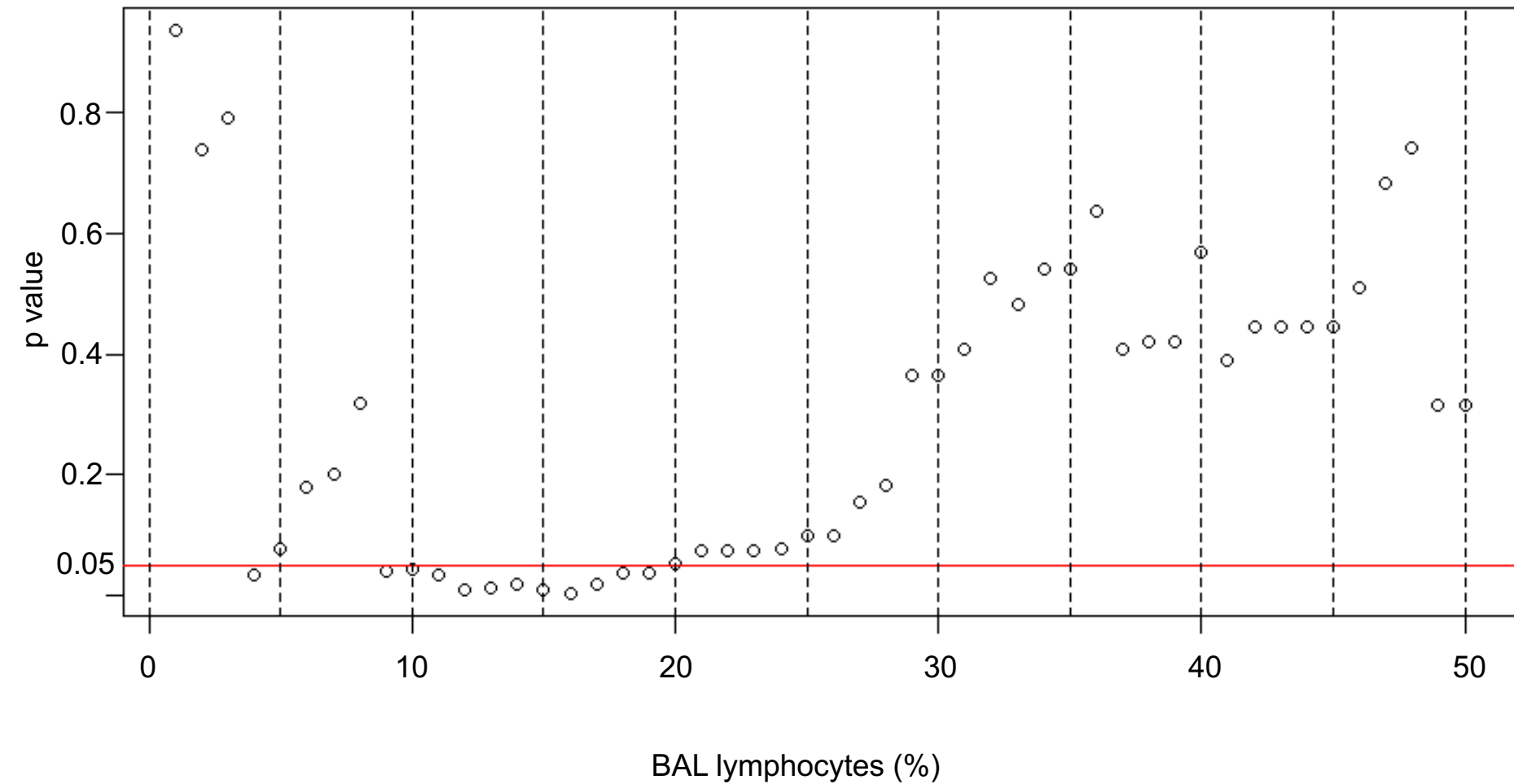

Supplement: Supplementary file 4 — Additional file 4: Fig. S2. Prognostic impact of BAL lymphocytosis on patients treated with anti-inflammatory drugs at each cut-off value [file 12931_2021_1726_MOESM4_ESM.pdf]

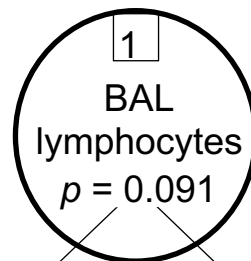

$\leq 16.6\%$

$> 16.6\%$

Node 2 (n = 114)

Node 3 (n = 72)

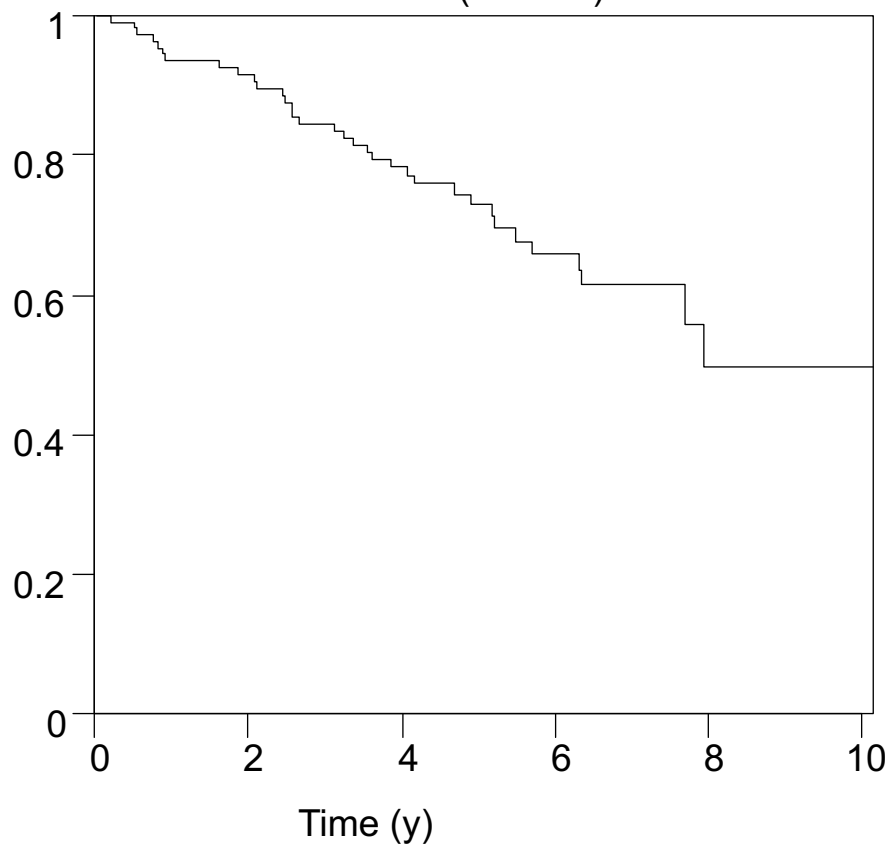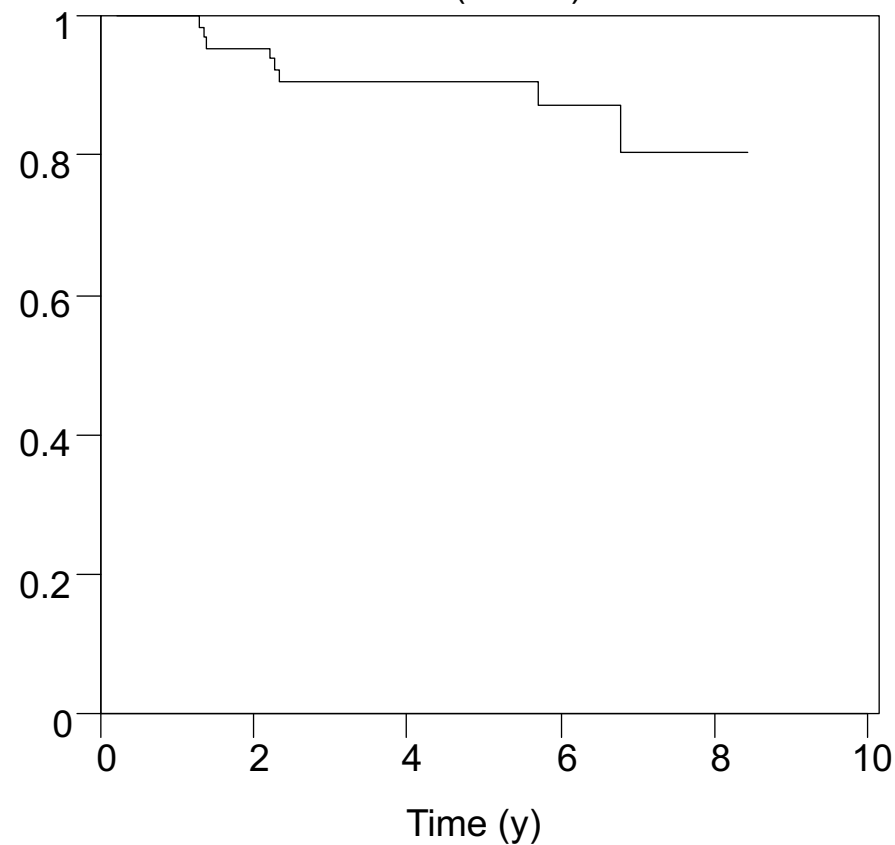

Supplement: Supplementary file 5 — Additional file 5: Fig. S3. CART analysis for predicting prognosis. [file 12931_2021_1726_MOESM5_ESM.pdf]
